# 381. Effect of Ceftriaxone+sulbactam+disodium EDTA Combination in Treatment of Complicated Urinary Tract Infections Caused by Metallo Beta-lactamase Producing Enterobacterales: An Open Label Randomized Controlled Trial

**DOI:** 10.1093/ofid/ofad500.451

**Published:** 2023-11-27

**Authors:** Subhashree Samantaray, Deepak Kumar, Gopal Krishana Bohra, Durga Shankar Meena, Ashwini Agarwal, Gautam Ram Chaudhary, Vibhor Tak, Ankur Sharma, M K Garg

**Affiliations:** AIIMS JODHPUR, Jodhpur, Rajasthan, India; All India Institute of Medical Sciences, Jodhpur (India), Jodhpur, Rajasthan, India; AIIMS Jodhpur, Jodhpur, Rajasthan, India; AIIMS, Jodhpur, Rajasthan, India; All India institute of medical sciences, Rajkot, Rajkot, Gujarat, India; All India institute of medical sciences, jodhpur, Jodhpur, Rajasthan, India; All India Institute of Medical Sciences, Jodhpur, Jodhpur, Rajasthan, India; All India Institute of Medical Sciences, Jodhpur, Jodhpur, Rajasthan, India; AIIMS, Jodhpur, Rajasthan, India

## Abstract

**Background:**

Ceftriaxone+sulbactam+disodium EDTA (CSE) is a novel antibiotic-adjuvant combination, approved by Drug Controller General of India (DCGI) against Extended Spectrum Beta-lactamase (ESBL) producing Gram-negative bacilli. Though randomized controlled trials (RCTs) have been conducted to prove efficacy of this combination against ESBL producers, there are only few in-vitro and retrospective studies supporting its role in treating Metallo-Beta Lactamase (MBL) producers. We conducted this RCT with an aim to find out the efficacy of CSE combination against the cUTI cases caused by MBL producing Enterobacterales.

Analysis of Primary End-points in Modified Intent-to-Treat Population

**Figure:**
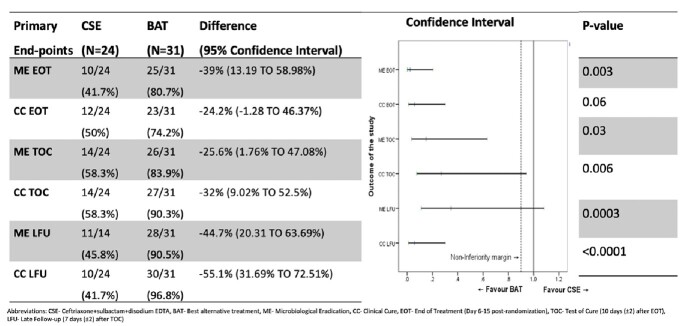

Table 1

**Methods:**

Adult patients aged ≥18 years meeting the USFDA criteria of cUTI, were randomized 1:1 to receive either intravenous CSE (1000 mg ceftriaxone/500 mg sulbactam/37 mg disodium EDTA) every 12 hours or best alternative treatment (BAT) as per hospital policy (injectable colistin or polymyxin B with or without meropenem or ceftazidime-avibactam with or without aztreonam). Primary objective was to show noninferiority of CSE to BAT at the test-of-cure (TOC) visit by a margin of 10%.

**Results:**

Of 66 randomized patients with cUTI growing MBL producing Enterobacterales in culture, 24 of 33 and 31 of 33 were treated with CSE and BAT, respectively. From outcome analysis, it was observed that the non-inferiority criteria for CSE to BAT was not met at pre-defined primary endpoints.(Table 1)

**Conclusion:**

Our findings do not support the use of CSE as a carbapenem-sparing treatment for cUTI patients caused by MBL producing Enterobacterales.

**Disclosures:**

**All Authors**: No reported disclosures

